# Combined miR-486 and GP88 (Progranulin) Serum Levels Are Suggested as Supportive Biomarkers for Therapy Decision in Elderly Prostate Cancer Patients

**DOI:** 10.3390/life12050732

**Published:** 2022-05-13

**Authors:** Alexander Fichte, Angela Neumann, Katrin Weigelt, Juan Guzman, Thilo Jansen, Julia Keinert, Ginette Serrero, Binbin Yue, Robert Stöhr, Thomas Greither, Arndt Hartmann, Bernd Wullich, Helge Taubert, Sven Wach, Verena Lieb

**Affiliations:** 1 Department of Urology and Pediatric Urology, Universitätsklinikum Erlangen, Friedrich-Alexander-Universität Erlangen-Nürnberg, 91054 Erlangen, Germany; alexander.fichte@uk-erlangen.de (A.F.); angela.neumann@uk-erlangen.de (A.N.); katrin.weigelt@uk-erlangen.de (K.W.); juan.guzman@uk-erlangen.de (J.G.); thilo.jansen@gmail.com (T.J.); julia.keinert@fau.de (J.K.); bernd.wullich@uk-erlangen.de (B.W.); sven.wach@uk-erlangen.de (S.W.); verena.lieb@uk-erlangen.de (V.L.); 2 Comprehensive Cancer Center Erlangen-EMN (CCC ER-EMN), 91054 Erlangen, Germany; robert.stoehr@uk-erlangen.de (R.S.); arndt.hartmann@uk-erlangen.de (A.H.); 3 A&G Pharmaceutical Inc., Columbia, MD 21045, USA; gserrero@agpharma.com; 4 Program in Oncology, University of Maryland Greenebaum Comprehensive Cancer Center, Baltimore, MD 21201, USA; byue@agpharma.com; 5 Department of Pathology, Universitätsklinikum Erlangen, Friedrich-Alexander-Universität, 91054 Erlangen, Germany; 6 Center for Reproductive Medicine and Andrology, Martin Luther University Halle-Wittenberg, 06120 Halle, Germany; thomas.greither@medizin.uni-halle.de

**Keywords:** progranulin, miRNAs, prostate cancer, serum level, predictive biomarker, age

## Abstract

Our study aimed to assess the applicability of miR-486 in combination with soluble GP88 protein as a diagnostic and/or predictive biomarker for prostate cancer (PCa) patients. miR-486 and GP88 levels in serum samples from 136 patients undergoing MRI-guided biopsy of the prostate were assessed by qRT–PCR and ELISA, respectively. Of these, 86 patients received a histologically confirmed diagnosis of PCa. Neither marker showed an association with the diagnosis of cancer. PCa patients were separated based on (i) treatment into patients with active surveillance or patients with any type of curative treatment and (ii) age into elderly (>68 years) patients and younger patients (≤68 years). In elderly patients (N = 41) with the intention of curative treatment at optimized cut-off values, significantly higher GP88 levels (*p* = 0.018) and lower miR-486 levels (*p* = 0.014) were observed. The total PSA level and ISUP biopsy grade were used in a baseline model for predicting definitive therapy. The baseline model exhibited an area under the curve (AUC) of 0.783 (*p* = 0.005). The addition of the serum biomarkers miR-486 and GP88 to the baseline model yielded an improved model with an AUC of 0.808 (*p* = 0.002). Altogether, combined miR-486 and GP88 serum levels are associated with and are therefore suggested as supportive biomarkers for therapy decisions, particularly in elderly PCa patients.

## 1. Introduction

Prostate cancer (PCa) remains a major cause of disease and mortality among men worldwide each year. In 2020, approximately 1.4 million men were diagnosed with PCa, and approximately 375,000 men died of PCa [1]. PCa is recognized as a genetically heterogeneous disease comprising a large variety of clinical courses ranging from indolent localized cancers that may never progress to rapidly progressing castration-resistant PCa (CRPC) [2]. It is clinically important to identify individuals needing and benefitting from early intervention while reducing the harms of ineffective treatments and/or overtreatment [3].

The temporal and spatial genomic heterogeneity of PCa together with different localizations and numbers of metastases exacerbate tissue-based molecular profiling in routine clinical practice. Therefore, blood-based liquid biopsies are a minimally invasive alternative that allows molecular analysis at the level of RNA, DNA, and protein in PCa [4]. Liquid biopsies have value as a source of prognostic, predictive, and response biomarkers in PCa [4,5,6]. In a liquid biopsy, circulating tumor cells, circulating nucleic acids, and exosomal vesicles are typically investigated [4]. However, liquid biopsy also allows further study of smaller or larger vesicles, protein–nucleic acid aggregates, or protein–protein aggregates for the presence and quantity of RNAs, DNAs, and proteins.

In serum, the prostate-specific antigen (PSA; kallikrein 3) protein is the most widely studied biomarker in PCa as either a single marker (total PSA or -2 pro PSA) or a combined marker in the Prostate Health Index (PHI: free PSA, total PSA, -2proPSA) or 4kscore (total PSA, free PSA, intact PSA, and human kallikrein 2) [7]. However, several other blood-based markers, such as human glandular kallikrein 2 (hK2), urokinase plasminogen activator (uPA) and its receptor (uPAR), and transforming growth factor-beta 1 (TGF-β1), interleukin-6 (IL-6) and its receptor (IL-6R), have been suggested [8].

MicroRNAs are small non-coding RNAs that have been described as tumor suppressors or oncogenes in different cancers, including prostate cancer [9,10,11]. In a previous study, we analyzed a selected set of seven miRNAs in combination with clinical and mpMRI information for PCa prediction and classification [12]. The addition of miR-486 and let-7c expression to the baseline model that exclusively included clinical parameters increased the predictive accuracy to identify clinically significant PCa [12].

Here, we additionally studied the progranulin (GP88) protein for its application as a serum biomarker in PCa. GP88 is an 88-kD glycoprotein described as an autocrine proliferation and survival factor for several cancer types [13,14]. GP88 can activate several tumor-related pathways, such as MEK/ERK [15], TNFR2 [16], AKT/PI3K [16,17], WNT [18], mTOR [19], and VEGF [20].

In a previous study, we found that low serum GP88 levels were more often detected in younger PCa patients, whereas high levels were noted in elderly PCa patients. A Gleason Score (GS) ≤ 6 at lower levels could be separated from GS7 or GS ≥ 8 patients. All PCa patients as well as younger PCa patients with lower GP88 serum levels showed better overall survival than those with higher levels [21].

In the present study, we analyzed miRNAs and GP88 serum levels in patients undergoing MRI-guided biopsy of the prostate. We identified that higher GP88 levels together with lower miR-486 levels were associated with definitive treatment in elderly PCa patients and could improve a baseline model for treatment prediction.

## 2. Materials and Methods

### 2.1. Study Population

A cohort of 136 consecutive patients with suspicion of PCa was recruited between January 2015 and July 2016 (Table 1). Patients were referred to the University Hospital Erlangen for targeted MRI-ultrasound fusion-guided prostate biopsy, which was complemented by systematic biopsy. Prostate mpMRIs were performed on 3T devices (Siemens Medical Solutions, Erlangen, Germany). Written informed consent was obtained before the biopsy, and the study was performed according to the Declaration of Helsinki. Ethical approval was provided by the ethics institutional review board of the University Hospital Erlangen (No. 3755, dated February 2008).

### 2.2. Blood Sampling and RNA Isolation

Before the biopsy, venous blood was drawn into coagulation tubes (Sarstedt, Nümbrecht, Germany) and further processed within two hours. Serum was prepared from the coagulated blood by centrifugation (2000× *g* for 10 min), and samples were stored in aliquots at −80 °C. Serum miRNAs were prepared from 200 µL of serum using the miRCURY RNA Isolation Kit for biofluids (Exiqon, Vedbaek, Denmark) according to the manufacturer’s recommendations.

### 2.3. Quantitative PCR

Quantification of miRNAs was conducted using miRCURY universal reverse transcription reagents and LNA-modified miRNA-specific primer sets specific to miR-21-5p, miR-141-3p, miR-210-3p, miR-320b, miR-375-3p, miR-486-5p and let-7c (Qiagen, Hilden, Germany) according to the manufacturer’s recommendations. Briefly, a constant volume of 6 µL of isolated serum RNA was reverse transcribed in a total volume of 20 µL using the miRCURY Universal cDNA Synthesis Kit. The resulting cDNA reaction was diluted 20-fold, and 4 µL was used as a template cDNA for the subsequent reactions. Quantitative PCRs were performed in a StepOne Plus real-time thermocycler (Thermo Scientific, Darmstadt, Germany) using LNA-modified primers and an SYBR green PCR mix (Qiagen). All reactions were measured in a final volume of 10 µL in triplicate. The thermal cycling conditions were chosen according to the manufacturer’s recommendations. For relative quantification, every sample was analyzed in parallel for the expression of specific miRNAs and the endogenous reference miRNA miR-16-5p. Baseline and threshold settings were constant across the complete experimental series. Reactions were regarded as valid when the threshold cycle Ct of miR-16-5p was within the range of 17–23. The relative expression levels were calculated by applying the ΔCt method [22] with all miRNA expression values reported as the ΔCt between reference and test miRNA. All reactions were performed blinded to the study endpoints.

### 2.4. GP88 ELISA

Serum GP88 levels were determined by a quantitative GP88 sandwich ELISA developed and manufactured by A&G Pharmaceutical, Inc. (Columbia, MD, USA), as described previously (Greither et al., 2018) using the anti-human GP88 6B3 monoclonal antibody as the coating antibody (10 μg/mL) and rabbit polyclonal GP88 antibody as the detection antibody. Standard samples (consisting of human GP88 at concentrations from 0 to 20 ng/mL) and patient and control samples were measured in duplicate. The ELISA reaction was measured based on absorbance with a TECAN Infinite M200 PRO (Tecan, Männedorf, Switzerland), and serum GP88 levels were quantified against the human GP88 standard curve.

### 2.5. Statistical Methods

Differences in the clinical factors and serum GP88 levels or miRNA expression values were analyzed using the Spearman Rho test, chi^2^ test, and Mann–Whitney test. Predictive modeling was performed using binomial generalized logistic regression modeling. Receiver operator characteristic (ROC) curves were calculated using the pROC package. For the estimation of the benefit of predictive models, we used the decision curve analysis method [23]. A *p* value of less than 0.05 was considered statistically significant. All statistical analyses were performed with the SPSS 28.0 software package (SPSS Inc., Chicago, IL, USA) and the R statistical framework Ver. 3.2.1 (the R foundation for statistical computing, Vienna, Austria).

## 3. Results

Of 136 consecutive patients with suspicion of PCa undergoing targeted MRI-TRUS guided biopsy, 86 patients received a histologically confirmed tumor diagnosis. The patients’ clinicopathological data are provided in Table 1. Of the PCa patients, 60 received a definitive curative treatment by radical prostatectomy, percutaneous radiation therapy, or brachytherapy, and 26 patients were treated by active surveillance. In addition, for all these PCa patients, GP88 levels and the expression levels of seven miRNAs (miR-141, miR-375, miR-21, miR-320, miR-210, let-7, and miR-486) were analyzed in the serum and correlated with the clinicopathological data.

### 3.1. GP88 Levels

GP88 levels were in the range of 25.9–99.4 ng/mL (median: 49.6 ng/mL). GP88 levels were not correlated with clinicopathological data. However, in patients where data for periprostatic invasion were reported (12 without vs. 6 with invasion), a negative correlation between GP88 level and periprostatic invasion was found in bivariate correlation (r_s_ = −0.727; *p* < 0.001; Spearman Rho test). In addition, the group with periprostatic invasion showed lower GP88 levels than the group without this invasion (*p* = 0.001; Mann–Whitney test).

Next, we studied whether GP88 levels at biopsy had a predictive value to distinguish between patients treated either by curative treatment or by active surveillance. The GP88 level of patients older than the median age of 68 years could distinguish between curative treatment and active surveillance with an area under the curve (AUC) value of 0.632 (*p* = 0.187) with a sensitivity of 79.3% and a specificity of 58.3%, but this difference was not significant. Moreover, the GP88 level did not show any potential for distinguishing between curative treatment and active surveillance in younger PCa patients (≤68 years). However, when applying the ROC optimized cut-off value of 45.03 for the elder PCa patients, patients with GP88 above the cut-off value were associated with a curative treatment (*p* = 0.018; Table 2).

### 3.2. miRNA Levels

The median ΔCT values for the seven miRNAs are provided in Table 1. The expression levels of the miRNAs were not correlated with the clinicopathological data.

Next, we were interested in whether miRNA levels could distinguish between curative treatment and active surveillance in PCa patients. In elderly patients (>68 years), miR-486 expression could distinguish between these two treatment options with an AUC of 0.670 (*p* = 0.091) with a sensitivity of 86.2 and a specificity of 50.0%, but this difference was not significant. Again, in younger PCa patients, miR-486 exhibited no potential for distinguishing between the two treatment options. However, when applying the ROC optimized cut-off value of 5.67 for the elder PCa patients, patients with miR-486 above the cut-off value were associated with a curative treatment (*p* = 0.014; Table 3). Here, a higher ΔCt value means a lower miR-486 expression.

### 3.3. Models for Distinction between Patients’ Treatment Intentions

**Figure 1:**
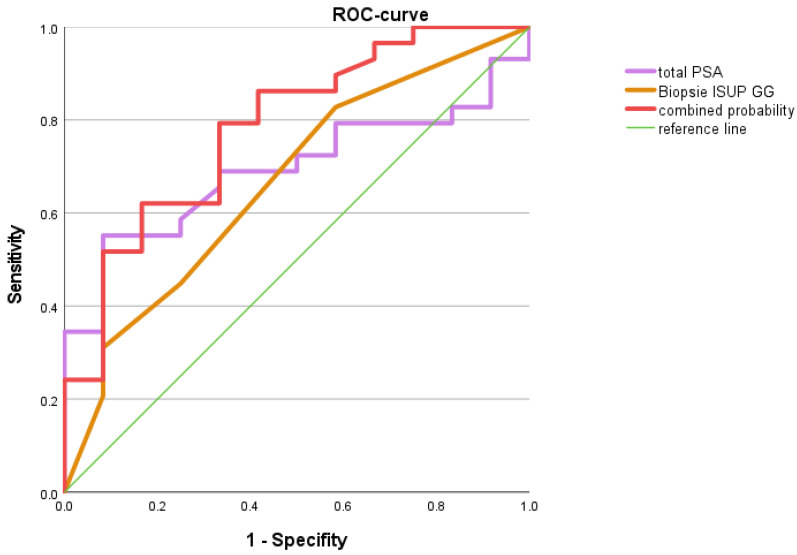
ROC analysis: Baseline model of total PSA and biopsy ISUP GG in elderly PCa patients. The baseline model of total PSA and ISUP GG allowed us to distinguish between the treatment intentions of curative treatment vs. active surveillance with an AUC of 0.783 (*p* = 0.005).

Regarding the decision between curative treatment or active surveillance, the two parameters, total PSA level and ISUP grade group at biopsy, were applied in a baseline model. As expected, both parameters could distinguish between the treatment intentions of curative treatment or active surveillance with an AUC of 0.783 (*p* = 0.005) with a sensitivity of 79.3% and a specificity of 66.7% in elderly PCa patients (Figure 1).

**Figure 2:**
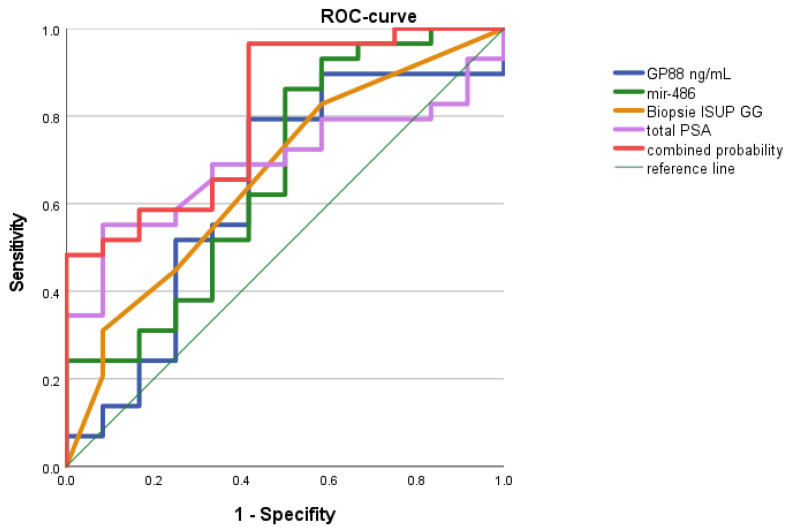
ROC analysis: Combined model of the baseline model (total PSA, biopsy ISUP GG) and experimental parameters (GP88 and miR-486) in elderly PCa patients. The combined model of total PSA, ISUP GG, GP88 levels, and miR-486 expression levels allowed us to distinguish between the treatment intentions of curative treatment vs. active surveillance with an AUC of 0.808 (*p* = 0.002).

We were interested in whether our two parameters, GP88 levels and miR-486 levels, could improve the baseline model. In the combined four-parameter model (PSA, ISUP grading group, GP88, miR-486) for the distinction between the treatment intentions of curative treatment vs. active surveillance, an AUC of 0.808 (*p* = 0.002) with a sensitivity of 96.6% and a specificity of 58.4% was obtained (Figure 2).

Altogether, the two experimental parameters, GP88 levels and miR-486 expression levels, improved the baseline model from an AUC of 0.783 to 0.808. The addition of these parameters to the model considerably increased the sensitivity from 79.3% to 96.6% but caused a rather small reduction in specificity from 66.7% to 58.4%.

**Figure 3:**
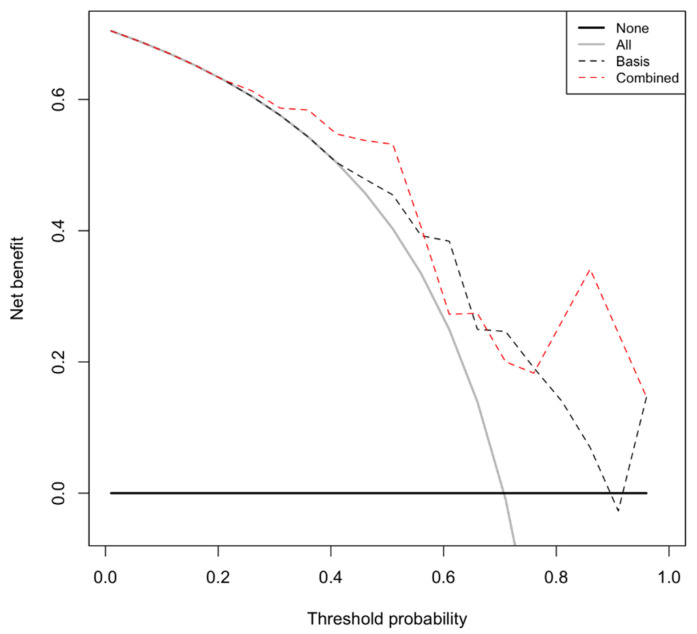
Decision curve analysis. The black dotted curve represents the baseline decision model that exclusively includes the clinical parameters (PSA at biopsy and ISUP GG). The red dotted curve represents the combined decision model that includes the baseline model together with the two parameters, namely, serum GP88 level and serum miR-486 level. The combined model (red dotted curve) provides a benefit over the extreme strategies of recommending a curative treatment in all patients (black curve) or recommending a curative treatment for no patient (black linear slope) and over the baseline model (black dotted curve) within a threshold probability range of 26% to 56%.

Finally, decision curve analysis was used to visualize the benefit of applying the combined predictive model. The combined four-parameter model provides a benefit over the extreme strategies of recommending a curative treatment in all patients or recommending a curative treatment for no patient. Most importantly, it also provides a benefit over the baseline model (PSA, ISUP grading group) within a threshold probability range of 26% to 56% (Figure 3; Supplementary Table S1).

## 4. Discussion

The difference in overall survival of PCa patients between radical prostatectomy versus observation for localized PCa is approximately 5% with a higher percentage of death in the observation group [3]. This notion again confirms, on the one hand, that an observation strategy, such as active surveillance, is a valid treatment option for low/intermediate risk PCa. However, on the other hand, lives could be saved when PCa patients who would benefit from a prostatectomy or another curative treatment could be identified as early as possible. Numerous biomarkers have been developed, but these biomarkers are not currently used in clinical practice for active surveillance [24,25]. Therefore, predictive markers to support therapy decisions between active surveillance and curative treatment in PCa are still eagerly awaited.

We previously reported on GP88 protein as a prognostic factor for PCa patients based on the detection of serum levels or in tumor tissue by IHC [21,26]. Furthermore, miR-486 serum levels supported the indication for MRI-ultrasound fusion-guided biopsy of the prostate in PCa patients with low-PI-RADS lesions [12]. Based on these results, we studied the levels of GP88 and miR-486 in the serum of PCa patients, correlated them with clinicopathological parameters, and investigated whether these parameters could be indicative of the therapy intention of active surveillance or definitive curative treatment in PCa. No correlation between serum levels of GP88 or miR-486 and clinicopathological parameters was noted with the exception of the GP88 serum protein level, which was inversely correlated with periprostatic invasion, but periprostatic invasion occurred in only a few cases. How could this unexpected finding be explained? Periprostatic adipose tissue (PPAT) covers the prostate anteriorly, and patients with more PPAT have worse cancer prognosis [27], leading to the view that PPAT-secreted factors stimulate tumorigenesis, particularly in obesity [28,29]. It is tempting to hypothesize that PPAT-associated growth factors may affect PCa cells but could also balance or even inhibit the expression of PCa-associated growth factors, such as GP88.

In elderly but not in younger PCa patients, the levels of GP88 (increased) and miR-486 (decreased) were helpful to distinguish between the decision of active surveillance or curative treatment, with an AUC of 0.632 or 0.670, respectively, but the differences between the decision intentions were not significant. Next, we considered the clinical applied parameters for the decision of active surveillance or curative treatment, i.e., prebiopsy PSA level and ISUP grading group. Both parameters were combined in a baseline model with an AUC of 0.783 (*p* = 0.005). However, after adding our molecular parameters, GP88 and miR-486, to the baseline model, we obtained a combined four-parameter model with an improved AUC of 0.808 (*p* = 0.002). To the best of our knowledge, this is the first study that showed a combined model involving a protein and a microRNA to help predict treatment intentions of active surveillance or curative treatment. However, several assays have been suggested for the identification of PCa patients eligible for active surveillance [24,30]. Several tests were able to improve the prediction of upgrading during the follow-up of active surveillance [24]. Among blood-based tests, the following tests are suggested for this treatment decision in plasma: 4k-panel and Stockholm-3. In addition, the following tests are suggested in serum: PHI, circulating prostate cells, caveolin-1, testosterone in men with hypogonadism, and a 3-microRNA score [24]. The 3-microRNA score including the miRNAs miRNA-24, miRNA-223, and miR-375, together with PSA serum levels had a predictive value (AUC of 0.757) for an upgrading of active surveillance patients [31]. In our study, only four patients out of the patients treated with active surveillance were upgraded during the follow-up, which did not allow a reasonable statistical analysis. The miR-375 included in the 3-miRNA score did not have a predictive value to distinguish between active surveillance and curative treatment in our study. However, in a previous study, we found that a combination of high miR-375 and high serum uPAR protein levels was an independent prognostic factor for the overall survival of PCa patients [32]. In our study, decreased miR-486 levels were indicative of the decision toward a definitive curative treatment of PCa patients. miR-486 has been reported as a diagnostic marker with higher expression levels in tumor tissues for several cancers (reviewed in [33]). However, in some cancers, it is described as a tumor suppressor, e.g., in hepatocellular carcinoma, esophageal squamous cell carcinoma, and chronic myeloid leukemia [34,35,36], and as an oncogene in other cancers, e.g., pancreatic ductal adenocarcinomas and glioblastoma multiforme [37,38]. Divergent findings have been reported in non-small-cell lung cancer (NSCLC). Chen et al. described miR-486 as a tumor suppressor in NSCLC progression [39]. Hu et al. reported it as a prognostic oncogene with higher expression in the serum of short survivors compared to long survivors [40]. Divergent findings have also been noted in prostate cancer. Yang et al. described it as an oncogenic miRNA. Specifically, this study noted that miR-486 is upregulated in tumor tissue compared to normal prostatic tissue, and inhibition of miR-486-5p reduced in vitro cell proliferation and in vivo tumorigenicity [41]. Song et al. found different miR-486-5p expression levels in tissues as follows: benign prostate hyperplasia (BPH) > high-grade PCa (GS > 7) > low-grade PCa (GS ≤ 7) [42]. Zhang reported that miR-486-5p suppresses prostate cancer metastasis by targeting Snail [43]. MiR-486 has been identified as a TP53-induced miRNA that can repress the mRNA expression of citron rho-interacting serine/threonine kinase (CIT) [44], which is associated with high expression and poor prognosis in PCa patients [45]. In our previous study, lower expression levels of miR-486 and let-7c together with clinical parameters in a model helped to better identify clinically significant PCa [12].

The predictive value of GP88 for active surveillance in PCa patients has not yet been studied. However, increased GP88 protein levels in tumor tissues or increased GP88 protein levels in serum have been associated with increased aggressiveness and poor long-term prognosis in different cancers, including PCa [14]. In addition, an increase in GP88 protein has been described in relation to different cancer treatment resistance mechanisms [46], especially in endocrine-related tumors, such as letrozole and tamoxifen resistance in breast cancer cells [20,47] and chemoresistance to cisplatin in ovarian cancer cell lines [48].

What is known about the regulation of GP88 in PCa? Monami et al. have shown that progranulin regulates the transformation of prostate cancer cells by promoting growth, migration, and invasion [49]. Tanimoto et al. described a negative feedback loop between sortilin and GP88 in PCa [50,51]. Sortilin is a cell receptor with a lysosomal sorting motif that is suggested to be involved in the endosomal/autophagosomal/lysosomal pathway [52]. Sortilin overexpression can negatively modulate AKT stability, downregulate AKT, and thereby affect AKT phosphorylation in PCa cell lines [51]. The AKT/PI3K pathway is involved in resistance to antiandrogen therapy, and androgen receptor inhibition is associated with an increase in AKT pathway activation in PCa [53]. Altogether, via the regulation of lysosomal degradation of sortilin, GP88 promotes the AKT/PI3K pathway.

The present study is limited due to its retrospective nature and our data analysis was performed after the treatment decision. Our patient cohort is rather early in the course of the disease and it covers the diagnosis and the first-line treatment. There are no castration-resistant PCa patients included. A survival analysis for overall and disease-free survival in our PCa study cohort was not meaningful due to the low number of events.

Given that frailty plays an important role in therapy decisions for elderly patients, e.g., the EAU guidelines recommend geriatric screening for all patients >70 years [54], our findings that the biomarkers GP88 and miR-486 were helpful in distinguishing between decision intentions for PCa patients >68 years may provide profound medical advice for this patient group, thus avoiding overtreatment and its comorbidities.

## 5. Conclusions

Combined serum miR-486 and GP88 levels are suggested as supportive biomarkers for therapy decisions in elderly PCa patients. A combination model incorporating these two markers together with the clinical parameters of prebiopsy PSA level and ISUP grading groups might support the decision between the treatment intentions of active surveillance or curative treatment in PCa patients.

## Figures and Tables

**Table 1:** Clinicopathological data and GP88/miRNA levels.

	N	N
	All/PCa Patients	Elderly PCa Patients
All suspected patients	136	56
Age range (median)	40–86 (67)	>68
PCa patients	86	41
Tumor-free patients	50	15
PCa patients Age range (median)	(48–86) 68	>68
ISUP grading of biopsy		
GG1 (GS6)	22	10
GG2 (GS7a)	28	15
GG3 (GS7b)	15	6
GG4 (GS8)	8	3
GG5 (GS9–10)	13	7
Treatment		
Curative treatments	60	29
Active surveillance	26	12
PSA at biopsy range (median)	0.6–112 (8.1)	2.2–85.0 (8.0)
<4 ng/mL	10	4
≥4 ng/mL	122	37
Unknown	4	0
GP88 level range (median)	25.9–99.4 (49.6)	25.9–96.8 (48.7)
ΔCt miR-141 range (median)	9.1–16.5 (12.9)	9.7–16.5 (12.8)
ΔCt miR-375 range (median)	7.8–15.7 (12.2)	8.2–15.1 (12.0)
ΔCt miR-21 range (median)	2.7–7.6 (4.1)	3.2–5.9 (4.0)
ΔCt miR-320 range (median)	7.5–11.9 (9.7)	8.5–11.9 (9.7)
ΔCt miR-210 range (median)	9.8–16.5 (11.6)	9.9–16.5 (11.6)
ΔCt let-7 range (median)	7.8–15.6 (9.6)	8.2–14.7 (9.8)
ΔCt miR-486 range (median)	4.7–7.3 (6.0)	5.2–7.3 (6.0)
Overall survival		
Alive	131	39
Deceased	5	2

**Table 2:** Cross table: Correlation between GP88 levels and therapy decision.

			GP88 Level in Elder PCa Patients		Sum
			≤45.3 ng/mL	>45.3 ng/mL	N
therapy decision	active surveillance	N	7	5	12
	curative treatment	N	6	23	29
Sum		N	13	28	41

*p* = 0.018 (chi^2^-test).

**Table 3:** Cross table: Correlation between ΔCt miR-486 expression and therapy decision.

			ΔCt miR486 Expression in Elder PCa Patients		Sum
			ΔCt ≤ 5.67	ΔCt > 5.67	N
therapy decision	active surveillance	N	6	6	12
	curative treatment	N	4	25	29
Sum		N	10	31	41

*p* = 0.014 (chi^2^-test).

## Data Availability

All data are available in the manuscript and the Supplementary Materials. Detailed datasets used and analyzed during the current study are available from the corresponding author on reasonable request.

## Supplementary Materials

The following are available online at https://www.mdpi.com/article/10.3390/life12050732/s1, Table S1: Values for decision curves.

## Abbreviations

AUC: area under the curve; CRPC: castration-resistant prostate cancer; EAU: European Association of Urology; ISUP: International Society of Urological Pathology; ISUP GG: ISUP grade group; mpMRI: multiparametric magnetic resonance imaging, PCa: prostate cancer.
